# Self-Healing Silver Nanowires and Reduced Graphene Oxide/Polyurethane Composite Film Based on the Diels–Alder Reaction under Infrared Radiation

**DOI:** 10.3390/membranes12040405

**Published:** 2022-04-06

**Authors:** Yi Wang, Zhimin Zhou, Jiali Chen, Sixing Li, Han Zheng, Jiaxin Lu, Shuyue Wang, Jiahao Zhang, Kaiwen Lin, Ke Wang, Yuehui Wang

**Affiliations:** Materials and Food Department, Zhongshan Institute, University of Electronic Science and Technology of China, Zhongshan 528402, China; wangyi@zsc.edu.cn (Y.W.); zzmzsedu@126.com (Z.Z.); chejiali0104@126.com (J.C.); 2019040801083@stu.zsc.edu.cn (S.L.); 2019040801059@stu.zsc.edu.cn (H.Z.); 2019040802023@stu.zsc.edu.cn (J.L.); 2019040802028@stu.zsc.edu.cn (S.W.); zjhzsedu@126.com (J.Z.); kevinlin1990@163.com (K.L.); wkzsedu@126.com (K.W.)

**Keywords:** silver nanowires, reduced graphene oxide, thermo-reversible Diels–Alder reaction polyurethane, self-healing, flexible conductive film

## Abstract

The hybrid composite of silver nanowires (AgNWs) and reduced graphene oxide (RGO) was synthesized in situ by an improved polyol–thermal method. The AgNWs-RGO with mass contents of 5–37 wt% was added into the thermo-reversible Diels–Alder reaction polyurethane (DA-PU) matrix with the AgNWs as the main conductor and the RGO as the auxiliary conductor to prepare self-healing composite conductive films. Further, the electrical conductivity, thermal conductivity, mechanical properties, infrared thermal response, and self-healing property of the composite film under infrared light irradiation were studied. The experimental results demonstrate that the AgNWs-RGO endows the composite film with good electrical and thermal conductivity and infrared thermal response ability, while the mechanical properties of the composite film decrease as the AgNWs-RGO mass content increases. The self-healing efficiency of the composite film is higher than that of the pure DA-PU under infrared light irradiation due to the good infrared photothermal response ability of the AgNWs-RGO. When the mass content of AgNWs-RGO in the composite film was 25 wt%, the AgNWs-RGO showed good dispersion in composite films, and the resistivity, thermal conductivity, and tensile strength of the composite film were 0.544 Ω·m, 0.3039 W·m^−1^·K^−1^, and 9.05 MPa, respectively. The infrared photothermal conversion temperature of the composite film is 158.5 °C (3450 lux for 1 min), and the infrared photothermal self-healing efficiency is 118% (3450 lux for 600 s). The AgNWs-RGO also improves the multiple self-healing ability of the composite film. The use of a high mass content of AgNWs-RGO in the composite film is beneficial in obtaining high multiple self-healing efficiencies. The first and the fifth infrared thermal self-healing efficiencies of the composite film with AgNWs-RGO of 35 wt% are 105% and 86%, respectively, and the resistivity of the composite film changes little and still maintains good conductivity.

## 1. Introduction

Over the last two decades, self-healing polymers including thermoplastic polyurethane (TPU) have attracted great attention due to the demands for improving the reliability, increasing energy efficiency, reducing waste, extending the service lifetime of material, and reducing the environmental impact of the materials and their potential applications in the fields of transportation, aerospace, electronics, and biomedical engineering [1,2,3,4,5]. As the name indicates, self-healing polymers are smart materials that can restore their mechanical properties or function after incurring damage, which is similar to the biological wound healing process in living organisms [6,7]. There are many strategies for developing self-healing polymers, which can be divided into autonomous and non-autonomous healing capabilities, with the aim of re-establishing microscale and macroscale cracks and regaining the original material properties and function; such strategies include microcapsules [8] or microfluid channel networks storing and releasing healing agent in the matrix [9] and dynamic covalent [10,11] or non-covalent bonds [12] introduced into polymers to form intrinsic self-healing polymers. Materials can heal themselves in either an autonomic or non-autonomic way, depending on whether human intervention is required during the healing process. The dynamic covalent bonds such as Diels–Alder (DA) bond reaction, dynamic disulfide metathesis, and ditelluride bonds have received increasing attention since the healing or reversibility can be governed by chemical bond interactions or activated by the input of external energy such as light, electromagnetic waves, humidity, or heat [13,14,15,16,17]. Though great progress has been made in the research of self-healing materials and techniques, there are still some critical problems that have restricted their applications, such as the low mechanical properties after healing, high cost, and time-consuming processes with low efficiency. Importantly, there is a contradiction between the self-healing efficiency and mechanical properties of polymers. So, it is a great challenge for us to obtain self-healing polymers and composites based on new materials and explore their potential applications as well as new healing methods.

Thermo-reversible DA-reaction polyurethane (DA-PU) of furan and maleimide moieties as a kind of intrinsic self-healing PU had been extensively implemented due to its high efficiency without a catalyst [18,19,20,21]. Upon thermal activation, the two moieties form a cyclo-adduct at low temperature, which reversibly regenerates the initial furan and maleimide groups at higher temperatures. At present, the DA-PU still has some problems such as poor mechanical properties, low self-healing efficiency, and rapid decline in self-healing efficiency with the increase in self-healing cycles [18,19,20,21,22,23,24,25]. In order to address these issues, scientists have made much progress in improving the performance of self-healing polymers. For example, in shape memory polymer fibers, polydopamine particles, graphene, and carbon nanotubes were introduced into the polymers as fillers [22,23,24,25,26]. Luan et al. prepared ultrafast near-infrared light responsive shape memory assisted self-healing polymer composites by introducing polydopamine particles (PDAPs) into polyurethane containing Diels–Alder bonds, which displayed enhanced mechanical properties, ultrafast near-infrared light responsive shape memory and self-healing properties [27]. Oh et al. prepared thermally self-healing graphene-nanoplate/polyurethane (GNP/PU) nanocomposites via a bulk in situ Diels–Alder (DA) reaction, in which the mechanical strength and chemical resistance were improved because of the crosslinking of the GNP in the polymer [28]. Zhang et al. reported that they prepared a self-healing flexible transparent conductor comprising a copper nanowire network and DA-PU via a transfer technique [29].

Recently, the introduction of nanomaterials such as carbon nanotubes, silver nanowires (AgNWs), and graphene (G) [30,31,32] into polymer materials to enhance the mechanical properties of materials and endow materials with new functions has been the subject of extensive research [15]. Two-dimensional (2D) graphene sheets play an active role in conductive polymer composites due to their high strength and Young’s modulus and extraordinary electrical and thermal conductivity. However, the graphene sheets tend to agglomerate in polymer matrices due to the intermolecular π–π stacking interaction. One-dimensional (1D) AgNWs are regarded as the best material for electrically and thermally conductive fillers due to their excellent intrinsic electrical and thermal conductivity and a high aspect ratio, which are beneficial in forming ideal electrically conductive networks at a relatively low content in polymer composites. However, AgNWs also tend to agglomerate in polymer matrices due to their large specific surface area [33].

Combining different types of fillers, such as conductive polymers/carbon nanotubes, graphene/carbon nanotubes, and AgNWs/graphene, has been a promising method to obtain lower percolation thresholds in composites with an enhanced electrical property [34,35]. In particular, hybrid fillers containing 1D and 2D materials can establish robust conductive pathways with lesser loading of fillers to generate a higher electrical conductivity while using a low amount of fillers because one type of filler bridges the gaps within the other type of filler. Additionally, the use of hybrid fillers yields a better dispersion of fillers in polymer matrices. At present, it has been reported that AgNWs and graphene were added into polymers in a composite or mixed manner to prepare composite materials [36,37,38,39]. AgNWs and graphene composites or mixtures can improve the dispersion of each other in the polymer matrix and the compatibility with the polymer matrix, and the synergistic enhancement effect of AgNWs and graphene can enhance the electrical conductivity, thermal conductivity, and mechanical properties of the composites, which greatly expands the applications of graphene and AgNWs [36,37,38,39]. In particular, the graphene and silver nanostructures as photothermal conversion reagents were used to trigger self-healing by absorbing infrared irradiation (IR) light and microwave radiation and converting it into thermal energy [40,41,42]. Kim and coworkers added modified graphene into PU to prepare self-healing nanocomposites with IR-triggered self-healing function [43]. Huang and coworkers prepared TPU with few-layer graphene and studied the self-healing performances of composites in response to microwave radiation [30]. Our group reported preparing graphene-TPU flexible conductive films by a blending method and systematically investigated the electrical, thermal, and self-healing properties of the graphene-TPU flexible conductive film in response to infrared light and electricity. A scratch on the composite film can be completely healed by using electricity or infrared light. The healing efficiency of the composite film healed under infrared light is higher than that of the composite film healed under electricity [44].

To the best of our knowledge, there are few reports on preparing thermo-reversible DA-PU composite film based on AgNWs and reduced graphene oxide (RGO). In this study, the hybrid composite of AgNWs-RGO was synthesized in situ by an improved polyol–thermal method. AgNWs-RGO was introduced into the thermo-reversible DA-PU matrix to prepare the self-healing composite conductive films with AgNWs as the main conductor and graphene as the auxiliary conductor. Further, the electrical and thermal conductivities, mechanical properties, infrared thermal effect, and self-healing property of the composite film under IR light were studied. Considering the excellent IR photothermal effect of the composite film, it is of great interest to investigate the self-healing performance of the composite under IR light.

## 2. Materials and Methods

### 2.1. Materials

Silver nitrate (AgNO_3_, >98 wt%) and ferric chloride hexahydrate (FeCl_3_·6H_2_O) were purchased from Aladdin (Shanghai) Reagent Co., Ltd., Shanghai, China. Polyvinylpyrrolidone (PVP, K30) was purchased from Sinopharm Chemical Reagent Co., Ltd., Shanghai, China. Ethylene glycol (EG) and N,N-dimethylformamide (DMF) were purchased from Tianjin Yongda Chemical Reagent Co., Ltd., Tianjin, China. DMF was dried over CaH_2_, and distilled prior to use. Acetone and absolute ethanol were purchased from Tianjin Baishi Chemical Co., Ltd., Tianjin, China. Poly(*tetrahydrofuran*) (PTMEG, Mn = 1000), 4,4-diphenylmethyl methane diisocyanate (MDI), furfurylamine (FA), and di-n-butylamine were purchased from Shanghai McLean Biochemical Co., Ltd., Shanghai, China; diphenylmethane bismaleimide (BMI) was purchased from Kema biochemical (Tianjin) Co., Ltd., Tianjin, China. Toluene was purchased from Dongguan Qiming Chemical Co., Ltd., Dongguan, Guangdong, China. Monolayer graphene oxide (GO, 3–5 μm, >98 wt%) powders were purchased from Chengdu Jiacai Technology Co., Ltd., Chengdu, China.

### 2.2. Preparation of Silver Nanowire–Reduced Graphene Oxide Composites

The typical preparation process is as follows: A certain amount of GO powders was added into a beaker containing 40 mL EG and subjected to ultrasonication at 300 W for 30 min. Then, 0.67950 g AgNO_3_ was added into the GO suspension and stirred with a glass rod until it was dissolved. Then, 0.06490 g FeCl_3_·6H_2_O was added into a beaker containing 40 mL EG and stirred with a glass rod until completely dissolved. Then, 01925 g PVP was added into a beaker containing 40 mL EG and stirred until completely dissolved, and then 1 mL FeCl_3_ solution was added into the PVP solution with a pipette gun and stirred for 5 min with a glass rod. According to the GO to AgNO_3_ mass ratio of 1:48.9, a certain amount of the mixed GO and AgNO_3_ solution was quickly mixed with the mixed PVP and FeCl_3_ solution under stirring, and then it was put into a 100 mL Teflon-lined autoclave tube. This tube was sealed and heated at 160 °C for 3 h, followed by natural cooling to room temperature. Composites of AgNWs and reduced graphene oxide (AgNWs-RGO) were purified by centrifuging at 3500 rpm for 20 min with acetone twice and then purified with absolute ethanol and centrifuged twice to obtain the AgNWs-RGO composites. It should be noted that the AgNWs-RGO composites were not dried used as slurry.

The effects of the mass ratio of GO to AgNO_3_ in the reaction solution, reaction temperature, and time on the morphology of the synthesized product were studied. The AgNWs-RGO composites used in the experiment were synthesized under the condition of a 1:48.9 mass ratio of GO to AgNO_3_ in the reaction solution.

### 2.3. Preparation of Prepolymer Thermally Reversible Diels–Alder (DA) Reaction Polyurethane

Prepolymer DA-PU was prepared using the conventional two-step method [18,19]. First, 30 g of PTMEG was added into a four-neck round-bottom flask that was filled with nitrogen, equipped with a mechanical stirrer, and heated to 60 °C. After the temperature was constant, 15.32 g MDI and 45.32 g DMF were added into the flask. The reaction was carried out at 60 °C under an argon atmosphere for 2.5 h until the isocyanate content in the reactant reached the theoretical endpoint (toluene di-n-butylamine titration). The reaction temperature was cooled to 0 °C with an ice–water bath. Then, 5.82 g FA dissolved in DMF (5.82 g) was added dropwise into the flask. After that, the reaction temperature was raised to 25 °C and the reaction was carried out for 20–30 min. The reaction progress was monitored with IR spectroscopy. Then, 10.9698 g BMI as a crosslinking agent and 42.03 g DMF were added into the flask. After that, the reaction temperature was raised to 60 °C and the reaction was carried out for 24 h, and thus the prepolymer DA-PU solution was obtained.

### 2.4. Preparation of AgNWs-RGO-DA-PU Composite Film

**Figure 1:**
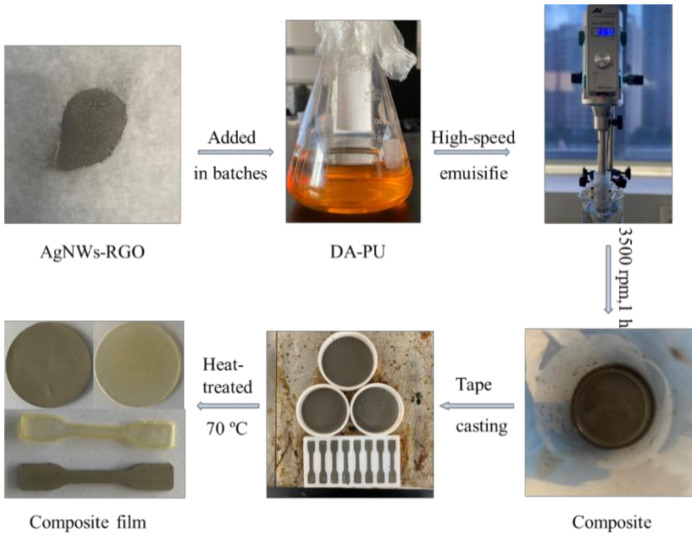
Schematic diagram of the fabrication process of AgNWs-RGO-DA-PU composite film.

A formulated amount of AgNWs-RGO slurry was added into a flask with DMF solution under vigorous stirring and ultrasonication at 300 W for 30 min. The AgNWs-RGO suspension was added into the formulated DA-PU solution with a 20 wt% mass content of DA-PU, and then the mixed solution was dispersed by a high-speed shear disperser at 3500 rpm·min^−1^ for 60 min. Finally, the mixed solution was poured into a Teflon mold. Most of the solvent and bubbles were removed at 50 °C on a heating plate, and then the solution was dried at 70 °C until constant weight to obtain the AgNWs-RGO-DA-PU composite film. The AgNWs-RGO-DA-PU composite film was peeled off for further testing. Figure 1 shows a schematic diagram of the fabrication process of the AgNWs-RGO-DA-PU composite film.

### 2.5. Characterization

Differential scanning calorimetry (DSC) was conducted via simultaneous differential thermal analysis (STA449F5, NETZSCH-Gertebau GmbH, Wittelsbacherstrabe, Selb, Germany). A scanning electron microscope (SEM, Zeiss sigma 500, Carl Zeiss, Germany) was used to investigate the microstructure of the composite film. The Raman spectrum was obtained using a Raman spectrometer (LabRAM HR Evolution, HORIBA JY, Paris, France). The resistance was measured using a four-point probe system (ST2253, Suzhou Jingge Electronics Co., Ltd., Suzhou, Zhejiang, China). An infrared thermal imager was used (UTI160G, range: −20 to 350 °C, accuracy: ±2 °C, UNI-T China Co., Ltd., Shenzhen, Guangdong, China). The thermal conductivity of the sample was measured using a DRL-III heat flow meter instrument (Xiangtan Xiangyi Instrument Co., Ltd., Xiangtan, Hunan, China) according to the standard ASTM D5470. Tensile strengths of the sample before and after healing were measured using a universal tensile testing machine (UTM500, Shenzhen Sansi Zongheng Technology Co., Ltd., Shenzhen, Guangdong, China). The extension rate was 50 mm/min. An infrared lamp (PHILIP PAR38E, 250 W, 0.76–5 μm, Royal Philips Electronics Co., Ltd., Suzhou, Jiangsu, China) was used as the light source. Before healing, a 5 mm scratch on the surface of the composite film was cut using a razor blade, and then the surface was exposed under IR light. After that, the tensile strengths of the healed samples were tested again. The healing efficiency was calculated as the ratio of tensile strength of the healed sample to that of the virgin specimen.

## 3. Results

### 3.1. Silver Nanowire–Reduced Graphene Oxide Composites

**Figure 2:**
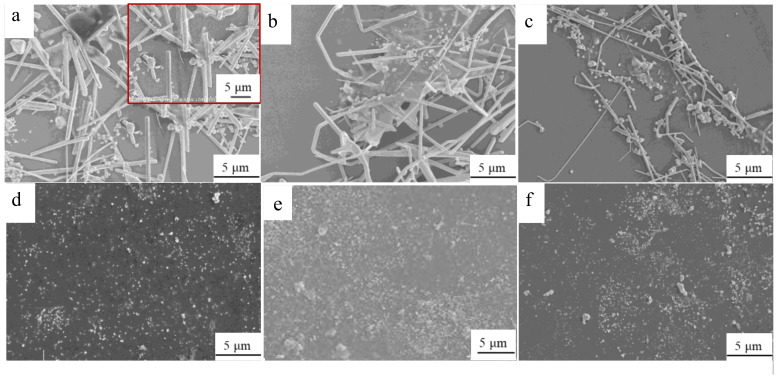
SEM images of products of the reaction of solutions with the GO:AgNO_3_ mass content ratios of 1:98.5 (**a**), 1:48.9 (**b**), 1:32.4 (**c**), 1:15.7 (**d**), 1:10.1 (**e**), and 1:7.3 (**f**) at 160 °C for 3 h. The inset is local magnification.

We synthesized a AgNWs-RGO hybrid composite in situ through a synchronous reduction of GO and silver ions using ethylene glycol as the chemical reduction agent at high temperature, GO and AgNO_3_ as raw materials, PVP as a surface modifier, ferric chloride as an inhibitor, and ethylene glycol as a solvent and reducing agent. We studied the effects of the mass ratio of GO to AgNO_3_ in the reaction solution, reaction temperature, and time on the morphology of the synthesized product. The experimental results show that the morphology of the synthesized product is related to the mass ratio of GO:AgNO_3_ in the reaction solution, reaction temperature, and time, and it is most affected by the mass ratio of GO:AgNO_3_ in the reaction solution. Figure 2 shows SEM images of the products synthesized with the GO:AgNO_3_ mass ratios in the reaction solution of 1:98.5 (Figure 2a), 1:48.9 (Figure 2b), 1:32.4 (Figure 2c), 1:15.7 (Figure 2d), 1:10.1 (Figure 2e), and 1:7.3 (Figure 2f) at 160 °C for 3 h. The inset in Figure 2a is local magnification. As shown in Figure 2, when the mass ratios of GO:AgNO_3_ in the reaction solution are 1:98.5 and 1:48.9, the products are mainly AgNWs-RGO composites, and graphene sheets cover the surface of AgNWs and fill the area between the AgNWs; when the mass ratio of GO:AgNO_3_ is 1:32.4, the amount of silver nanoparticles in the product increases significantly, and the AgNWs become thin; when the mass ratios of GO:AgNO_3_ are 1:15.7, 1:10.1, and 1:7.3, the products are hybrid composites of silver nanoparticles and graphene sheets. As the reaction temperature increases, the amount of the silver nanoparticles in the product increases, and the size of AgNWs decreases. As the reaction time increases, the size of AgNWs in the product decreases, and the number of AgNWs increases. More information about SEM images of the products synthesized under different reaction temperatures and times are shown in Figures S1 and S2 of the Supplementary Materials.

Some researchers obtained AgNWs-RGO hybrid composites by using mechanical mixing of the AgNW suspension and graphene suspension or the AgNW suspension and graphene oxide dispersion before reduction with hydrazine hydrate [45,46]. Graphene or GO acts as a dispersion agent to prevent the agglomeration of AgNWs. As seen in our experiment, because the GO sheets containing a large number of negatively charged oxygen-containing functional groups (such as hydroxyl, carboxyl, and carbonyl groups) are negatively charged, the GO sheets adsorb a large amount of Ag^+^ and Fe^3+^ around them through electrostatic interaction. During the solvothermal reaction, GO and Ag^+^ are reduced by ethylene glycol, and the silver atoms (Ag^0^) aggregate on the GO sheet. During the reduction process, the Fe^2+^ generated by the reduction of Cl^−^ and Fe^3+^ inhibits the nucleation rate of Ag^0^, forming a double 10-sided twinned Ag crystal nucleus on the graphene sheet; after that, PVP is selectively adsorbed on the crystal plane of <100> of nucleation, which promotes the deposition of silver atoms on the crystal plane of <111> of nucleation and gradually grows into wire. Meanwhile, during the solvothermal reaction, GO is also reduced synchronously, bridging the AgNWs.

**Figure 3:**
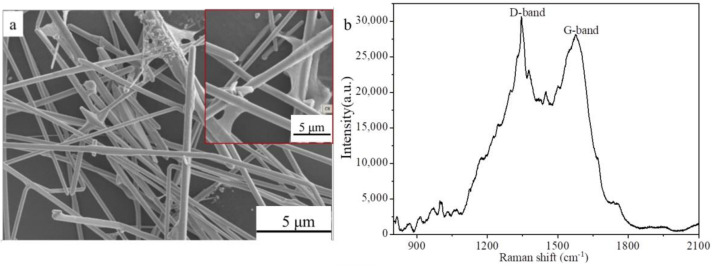
SEM image (**a**) and Raman spectrum (**b**) of the AgNWs-RGO composites. The inset is local magnification.

The products synthesized with the mass ratio of GO:AgNO_3_ of 1:48.9 in the reaction solution were further purified to remove silver nanoparticles by centrifugation at 2000 rpm for 30 min. Figure 3 displays the SEM image (Figure 3a) and Raman spectrum (Figure 3b) of the purified products. The inset in Figure 3a is local magnification. The hybrid composites of AgNWs-RGO are clearly observed. and graphene sheets cover and overlap the surface of the AgNWs (the inset in Figure 3a). The AgNW diameter is about 200–300 nm, and the AgNW length is about 20–40 μm. The D peak at 1350 cm^−1^ and the G peak at 1600 cm^−1^ in the Raman spectrum are observed, and the D peak intensity (I_D_) is greater than the G peak intensity (I_G_), indicating that GO sheets are successfully reduced to graphene by EG. However, the peak position of D peak indicates that the edges of graphene sheet are defective [47].

### 3.2. Thermo-Reversible DA-Reaction Polyurethane

The preparation of thermo-reversible DA-reaction polyurethane (DA-PU) of furan and maleimide moieties has been extensively studied [18,19,20,21,22,23,24,25]. Here, we used the same strategy to synthesize the DA-PU solution. The reaction progress was monitored with IR spectroscopy as shown in Figures S3 and S4 of the Supplementary Materials. The molecular weight of DA-PU was measured using a gel permeation chromatograph, as shown in Figure S5 of the Supplementary Materials. The number average molecular weight (M_n_) and weight average molecular weight (Mw) of DA-PU are 12,421 and 37,625, respectively, indicating that DA-PU is a nonamer structure polymerized by three BMI and six PTMEG-MDI-FA molecules [18,19]. The ratio of Mw to Mn is about 3, which indicates that the molecular weight distribution of DA-PU is relatively uniform [19].

**Figure 4:**
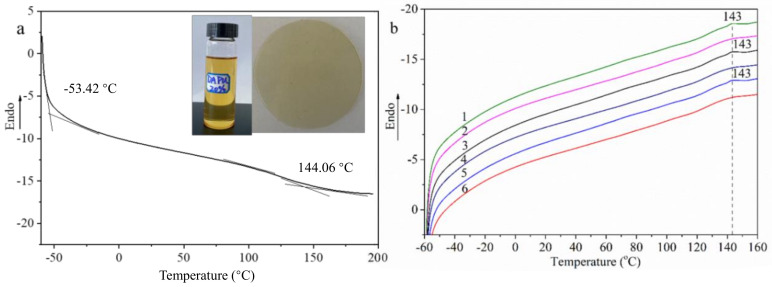
DSC curve of DA-PU (**a**) and DSC curves of the reversibility test of the sample (**b**); 1–6 in Figure 3b are (1) DA0, (2) rDA1, (3) DA1, (4) rDA2, (5) DA2, and (6) rDA3. The insets in Figure 3a are prepolymer DA-PU of 20 wt% and cured DA-PU film.

In order to discuss the thermal reversibility of DA-PU, the DA-PU film was subjected to a cyclic heat-treatment process. The DA-PU film (marked as DA0) was put into an oven at 140 °C for 30 min and then quenched with liquid nitrogen to obtain rDA1, which was heated at 60 °C for 24 h and then quenched to obtain DA1. The above process was repeated twice to obtain rDA2, DA2, and rDA3. Figure 4 shows the DSC curve of DA-PU (Figure 4a) and the DSC curves of the reversibility test of the sample (Figure 4b); 1–6 in Figure 4b are (1) DA0, (2) rDA1, (3) DA1, (4) rDA2, (5) DA2, and (6) rDA3. The insets in Figure 2a are prepolymer DA-PU of 20 wt% and cured DA-PU film. As shown in Figure 4a, the glass transition temperature and the crystalline melting of DA-PU are at around −53.42 °C and 144.06 °C, respectively. There is a weak endothermic peak at 143 °C on the DSC curve of DA0 and no peak on the DSC curve of rDA1, indicating that the DA bond in DA-PU has broken and resulted in a DA monomer after heat treatment at 140 °C. The DSC curve of DA1 shows the endothermic peak of the DA bond near 143 °C, indicating that the DA monomer in rDA1 regenerates the DA bond after heat treatment at 60 °C. Similarly, the disappearance of the DA bond endothermic peak in the DSC curves of rDA2 and rDA3 and the recurrence of the DA endothermic peak in the DA2 curve all show that DA reaction and retro-DA reaction exist in the DA-PU structure, meaning that DA-PU has good thermal reversibility.

### 3.3. Microstructures of AgNWs-RGO-DA-PU Composite Film

**Figure 5:**
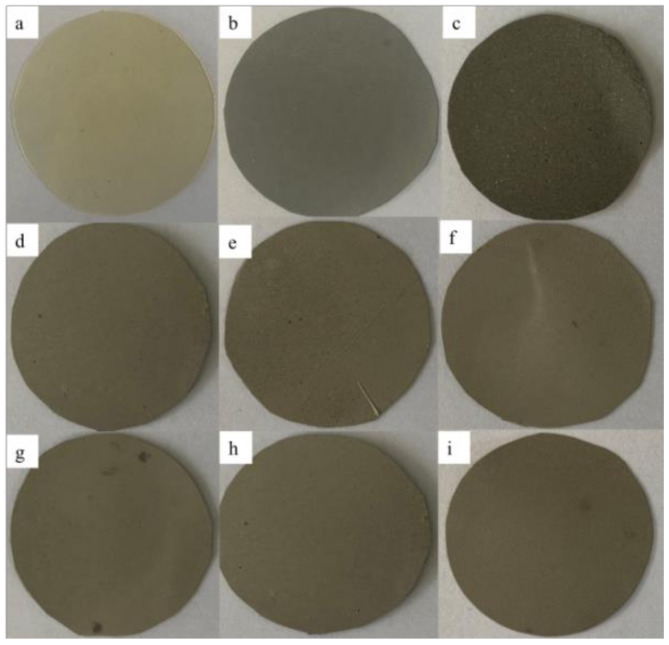
Photographs of the AgNWs-RGO-DA-PU composite films with the AgNWs-RGO mass contents of 0 wt% (**a**), 5 wt% (**b**), 10 wt% (**c**), 15 wt% (**d**), 20 wt% (**e**), 25 wt% (**f**), 30 wt% (**g**), 35 wt% (**h**), and 37 wt% (**i**).

AgNWs-RGO-DA-PU composite films with the AgNWs-RGO mass contents of 5 wt%, 10 wt%, 15 wt%, 20 wt%, 25 wt%, 30 wt%, 35 wt%, and 37 wt% were prepared. Figure 5 shows the photos of the pure DA-PU film and the composite films. As can be seen in Figure 5, the pure DA-PU film is light yellow and translucent, while the composite films containing AgNWs-RGO are gray, and the film color deepens with the increase in AgNWs-RGO mass content.

**Figure 6:**
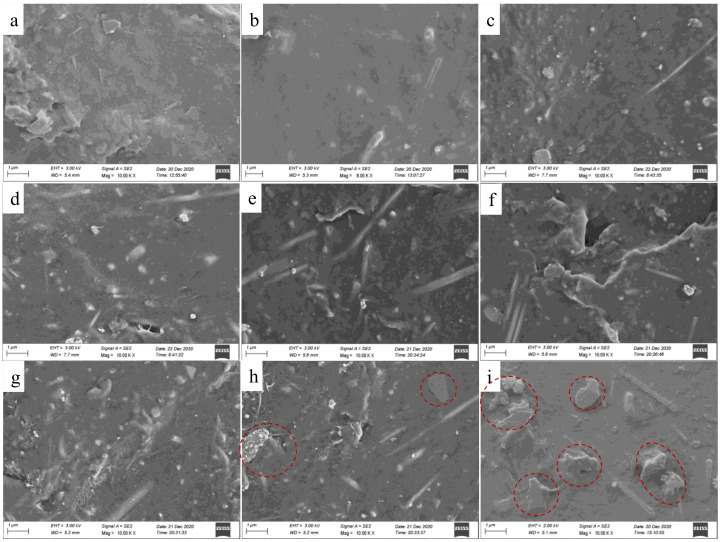
SEM images of cross-section morphology of the composite films with the AgNWs-RGO mass contents of 0 wt% (**a**), 5 wt% (**b**), 10 wt% (**c**), 15 wt% (**d**), 20 wt% (**e**), 25 wt% (**f**), 30 wt% (**g**), 35 wt% (**h**), and 37 wt% (**i**).

Figure 6 shows SEM images of the cross-section morphology of the pure DA-PU film (Figure 6a) and the composite films with the AgNWs-RGO mass contents of 5 wt% (Figure 6b), 10 wt% (Figure 6c), 15 wt% (Figure 6d), 20 wt% (Figure 6e), 25 wt% (Figure 6f), 30 wt% (Figure 6g), 35 wt% (Figure 6h), and 37 wt% (Figure 6i). The cross-section view of the pure UA-PU shows that it is smooth. The composite film containing 5 wt% AgNWs-RGO shows mainly cured DA-PU, and a very small amount of AgNWs was observed. The cross-section view surfaces of the composite films containing 10 wt% and 15 wt% AgNWs-RGO become rough, and the AgNWs and granular structures can be observed. The cross-section of the composite film containing 20 wt% AgNWs-RGO clearly shows that the AgNWs-RGO is embedded into the cured DA-PU matrix. As the mass contents of AgNWs-RGO increase to 35 wt% and 37 wt%, massive aggregates (the areas in the dotted circle) are observed, indicating that the dispersion of the high mass content AgNWs-RGO and the compatibility between AgNWs-RGO and matrix are poor. In our experiment, we found that the DA-PU slurry with 37 wt% AgNWs-RGO has poor mobility and is hard to cast into a uniform film. It should be pointed out that because the content of the graphene in the AgNWs-RGO is low and the graphene sheet is very thin, graphene cannot be observed in the SEM images.

### 3.4. Electrical and Thermal Properties of AgNWs-RGO-DA-PU Composite Film

**Figure 7:**
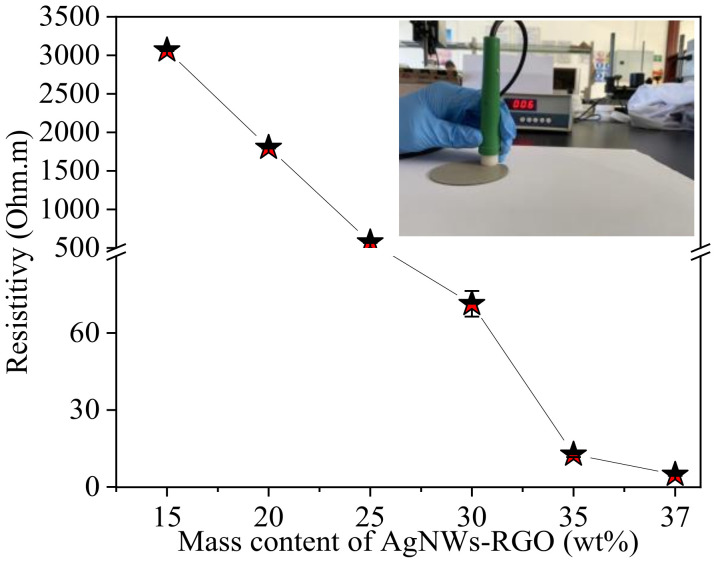
Resistivities of the composite films with different AgNWs-RGO mass contents. The inset is a photograph of the testing sample.

Figure 7 shows the resistivity of AgNWs-RGO-DA-PU composite films with different mass contents of AgNWs-RGO. The inset is a photo of the testing sample. In our experiment, we found that the resistance of the composite films containing AgNWs-RGO at 5 wt% and 10 wt% cannot be tested. This is because the AgNWs-RGO mass content in the composite film is too low to form effective conductive networks. The resistivity of the composite film containing 15 wt% AgNWs-RGO is 3.066 Ω·m, indicating that the AgNWs-RGO mass content in the composite film exceeds the percolation threshold and the AgNWs-RGO structures overlap with each other to form effective conductive networks [43]. With the increase in the AgNWs-RGO mass content, the resistivity of the composite film decreases gradually, and the resistivities of the composite films with the AgNWs-RGO mass contents of 25 wt%, 30 wt%, 35 wt%, and 37 wt% are 0.544 Ω·m, 0.067 Ω·m, 0.013 Ω·m, and 0.005 Ω·m, respectively. In the process of heat treatment, with the evaporation of the solvent, the DA reaction between furan-terminated prepolymer and BMI takes place and long PU-DA chains are generated, so covalent cross-linking structures are gradually formed. The curing and shrinkage of the DA-PU promote the proximity, overlap, and stacking of the AgNWs-RGO, which provides robust and effective conductive paths. The higher the content of AgNWs-RGO, the more effective conductive paths, and the better the conductivity of the composite film. However, when the effective conductive networks formed reach a certain amount, the enhancement effect of increasing the mass content of AgNWs-RGO on the electrical conductivity is not obvious. On the contrary, due to the difficulty in dispersing the AgNWs-RGO in the prepolymer DA-PU solution, the AgNWs-RGO agglomerates are formed in the high AgNWs-RGO mass content DA-PU solution; as a result, it is hard to cast a uniform film due to the poor fluidity of slurry.

**Figure 8:**
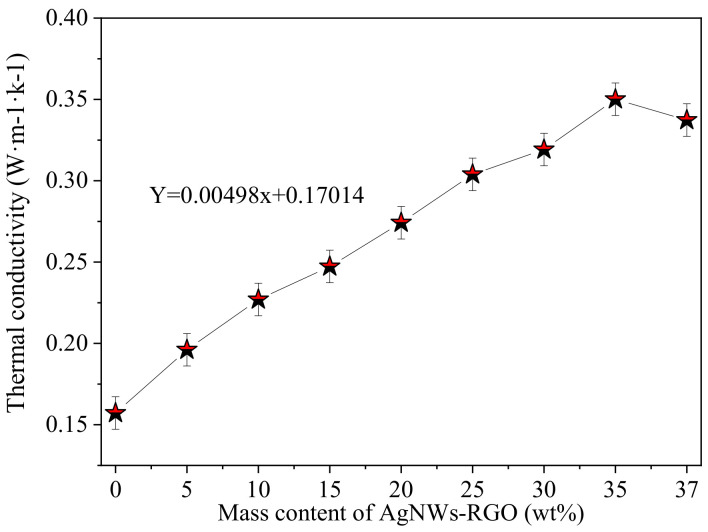
Thermal conductivity of composite films with different AgNWs-RGO mass contents.

It should be pointed out that the in situ synthesized AgNWs-RGO is used as a conductive filler; the RGO sheets coat and overlap between the AgNWs and fill in the gaps of the AgNW networks, enhancing the conductive interconnection between the AgNWs. Moreover, the hydroxyl and carboxyl groups on the edge of the RGO sheets also improve the dispersibility of the AgNWs and RGO in the DA-PU matrix. In fact, we also tried to prepare the conductive film by adding pure AgNWs as conductive fillers into the DA-PU matrix. However, it was found in the experiment that when the dispersed AgNW suspension was added into the prepolymer DA-PU solution, the AgNWs agglomerated and could not be stirred or dispersed. Both AgNWs and RGO are materials with good electrical and thermal conductivity. The AgNWs-RGO in the polymer matrix can impart electrical conductivity and thermal conductivity to the polymer. The thermal conductivity of the composite film is another important property, so we measured the thermal conductivity of the composite films with different mass contents of AgNWs-RGO, as shown in Figure 8. The thermal conductivity of the pure DA-PU is about 0.1572 W·m^−1^·K^−1^. As the mass content of AgNWs-RGO increases from 5 wt% to 35 wt%, the thermal conductivity of the composite film increases approximately linearly to 0.3501 W·m^−1^·K^−1^, increasing by a factor of 2.2. After that, the thermal conductivity of the composite film containing 37 wt% AgNWs-RGO decreases. As previously stated, the AgNWs-RGO is added to the DA-PU matrix to form thermal conductivity networks. Due to the synergistic effect between AgNWs and RGO, the thermal conductivity of the film is significantly improved. However, when the AgNWs-RGO mass content in the composite film is too high, the overlap and interconnection between AgNWs-RGO structures are deteriorated due to AgNWs-RGO agglomeration, resulting in a decrease in the number of heat conduction paths and thermal conductivity.

### 3.5. IR Thermal Response Performances of AgNWs-RGO-DA-PU Composite Film

**Figure 9:**
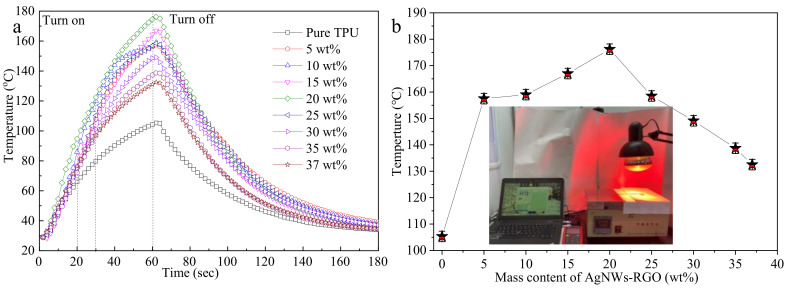
Temperature of the composite films containing different AgNWs-RGO mass contents under IR light irradiation (**a**) and the temperature of the composite film irradiated for 1 min (**b**). The inset in Figure 6b is a photograph of the testing sample.

Light is the most popular power mode and can heat materials in space and time, and the light-induced temperature rise can be controlled by adjusting the light intensity. Near-infrared light can effectively penetrate through materials, which makes it an ideal choice for remote stimulus control [40,41]. It has been reported that graphene sheets and AgNWs have good absorption characteristics for infrared irradiation (IR) light [40,41,48]. Here, we investigated the IR thermal response of the composite film. Figure 9 shows the temperatures of composite films containing different AgNWs-RGO mass contents as a function of the irradiation time under IR light (Figure 9a) and the temperature of the composite film irradiated for 1 min (Figure 9b). The inset in Figure 9b is a photograph of the testing sample. A circular sample with a diameter of 30 mm was placed under IR light with the intensity of 3450 lux for 1 min and cooled to room temperature naturally. The temperature sensor was used to detect the temperature of the sample. As shown in Figure 9a, the temperatures of the pure DA-PU film and the composite film increase linearly with time in 20 s and 30 s, respectively, after turning on the IR light. Thereafter, the temperature of the film increases slowly with the increase in IR time. After irradiation for 60 s, the temperatures of the pure DA-PU film and the composite films with the AgNWs-RGO contents of 5 wt%, 10 wt%, 15 wt%, 20 wt%, 25 wt%, 30 wt%, 35 wt%, and 37 wt% reach 105.3 °C, 157.5 °C, 159.0 °C, 167.0 °C, 176.2 °C, 158.5 °C, 149.1 °C, 138.7 °C, and 132.5 °C, respectively. The temperature of the composite film with 20 wt% AgNWs-RGO is maximal. After turning off the IR light, the temperature of the composite films gradually decreases to below 30 °C in 120 s. The above experimental results reveal that the AgNWs-RGO remarkably improves the IR response performance of the DA-PU film.

AgNWs and RGO have a strong ability to absorb IR light due to the unique nanoscale structure of AgNWs and the two-dimensional conjugated structure of RGO. When IR light is irradiated on the surface of the composite film, AgNWs, RGO ,and DA-PU absorb the infrared radiation energy, convert it into thermal energy, and transfer the thermal energy to the polymer matrix through the thermal network of AgNWs-RGO, causing the composite film to heat quickly. When the AgNWs-RGO mass content in the composite film is within a certain range, as the AgNWs-RGO content increases, the numbers of energy conversion centers and heat transfer pathways in the composite film increase. Therefore, the temperature of the composite film gradually rises under IR light irradiation for 1 min. However, when the mass content of AgNWs-RGO in the composite film is too high, AgNWs-RGO has difficulty dispersing in the DA-PU matrix and easily forms agglomerates, which affect the absorption of infrared radiation and reduce the heat conduction pathways in the composite film, resulting in a decrease in the temperature of the composite film.

### 3.6. Mechanical Performances of AgNWs-RGO-DA-PU Composite Film

**Figure 10:**
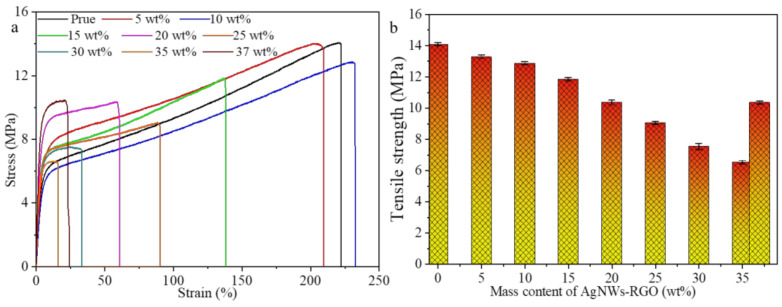
Stress–strain curves (**a**) and tensile strength (**b**) of the composite films with different AgNWs-RGO contents.

Figure 10 shows the stress–strain curves of the composite films with different AgNWs-RGO mass contents. The corresponding mechanical properties are listed in Table 1. It can be seen from Figure 10 and Table 1 that the tensile strength of the pure DA-PU film is higher than that of DA-PU with the addition of the AgNWs-RGO. As the AgNWs-RGO content increases from 0 wt% to 35 wt%, the tensile strength of the composite film decreases from 14.08 to 6.63 MPa. When the AgNWs-RGO content further increases to 37 wt%, the tensile strength of the composite film increases to 10.55 MPa. As shown in Table 1, the Young’s modulus of the composite film is higher than that of the pure DA-PU film. As the AgNWs-RGO content increases from 0 to 20 wt%, the Young’s modulus of the composite film increases from 19.30 to 238.91 MPa. As the AgNWs-RGO content increases to 37 wt%, the Young’s modulus increases to 246.40 MPa. As the AgNWs-RGO content increases from 0 wt% to 35 wt%, the fracture strain of the composite film decreases from 221.94% to 15.85 MPa, except that the fracture strain of the composite films with AgNWs-RGO content of 10 wt% increases slightly. It is noteworthy that when the content of the AgNWs-RGO in the composite film reaches and exceeds 15 wt%, the composite film displays a significant increase in Young’s modulus and a significant decrease in fracture strain compared to those of the pure DA-PU film, indicating that the AgNWs-RGO decreases the flexibility of the DA-PU film; however, the flexibility of the composite films with a low content of the AgNWs-RGO can be maintained. It is reported that the low content of graphene, GO, carbon nanotubes, or other inorganic fillers as reinforcement filler can enhance the mechanical properties of the polymer composites [33,45] due to the efficient load transfer between the filler and the polymer matrix resulting from the chemical bonding and the uniform dispersion of low content filler in the polymer matrix. However, our experimental results reveal that the AgNWs-RGO did not enhance the tensile strength and fracture strain of the composite film. This can be attributed to the fact that the composite film with higher AgNWs-RGO mass content (compared with graphene, GO, or carbon nanotube mass content in the polymers reported, the AgNWs-RGO mass content in DA-PU in our experiment is high) exhibits stress concentration on the AgNWs-RGO, which leads to the breaking of the composite film during stretching. Meanwhile, a large amount of the AgNWs-RGO in the composite also interferes with the orientation of the DA-PU molecular chain, preventing the composite from maintaining the intrinsic flexibility of the DA-PU matrix [44,45,46,47,48,49,50,51,52]. In addition, due to the high content of the AgNWs-RGO in the composite, we believe that the mechanical properties of the composite film are determined by the synergistic effect of the DA-PU matrix and the AgNWs-RGO fillers. When the AgNWs-RGO loading is low, the mechanical properties of the DA-PU matrix are dominant, while the fillers degrade the mechanical properties of the composite. When the AgNWs-RGO loading reaches a certain amount, the AgNWs-RGO with good flexibility can enhance the mechanical properties of the composite. In addition to the reasons described above, the agglomeration of the AgNWs-RGO in the DA-PU matrix and the weak binding between the AgNWs-RGO and the DA-PU matrix also lead to the degradation of the mechanical properties of the composite film [53].

### 3.7. Self-Healing of AgNWs-RGO-DA-PU Composite Film

**Figure 11:**
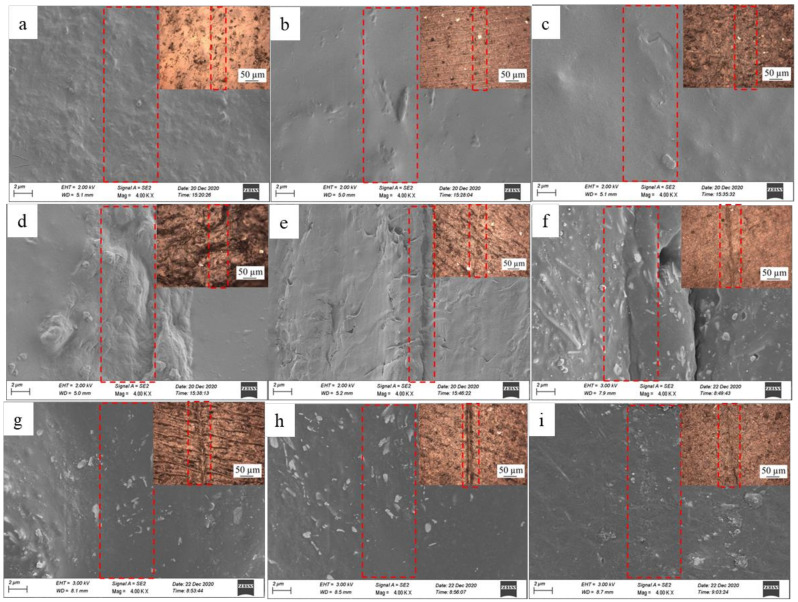
SEM images of self-healing composite films with the AgNWs-RGO mass contents of 0 wt% (**a**), 5 wt% (**b**), 10 wt% (**c**), 15 wt% (**d**), 20 wt% (**e**), 25 wt% (**f**), 30 wt% (**g**), 35 wt% (**h**), and 37 wt% (**i**). The insets are optical microscope photographs.

**Figure 12:**
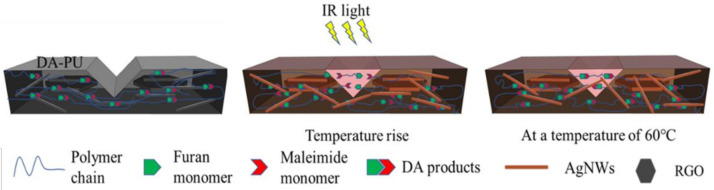
Schematic diagram of the self-healing of the AgNWs-RGO-DA-PU composite film under IR light.

The purpose of the research and development of thermally reversible DA-PU is to develop a kind of smart PU; as the material is damaged due to the occurrence and propagation of micro/macrocracks on the interior or surface of the material, the self-healing of the microcracks can be achieved through heat treatment, and then the performances of the material can be restored. To investigate the influence of the AgNWs-RGO on the self-healing performance of the composite film, a 0.5 mm scratch was cut on the surface of the sample, and then the sample was put under IR light with 3450 lux for a period of time; when the scratch disappeared as determined by naked eye observation, the IR light was turned off, the time was recorded, and the sample was put into an oven at 60 °C for 20 min. The experimental results reveal that the scratches of the pure DA-PU and the composite films containing the AgNWs-RGO mass contents of 5 wt%, 10 wt%, 15 wt%, 20 wt%, 25 wt%, 30 wt%, 35 wt%, and 37 wt% under IR light irradiation disappear after 300 s, 90 s, 100 s, 460 s, 540 s, 600 s, 870 s, 1205 s, and 1515 s, respectively, indicating that self-healing behavior occurs. Figure 11 shows SEM images of the self-healing composite films with different AgNWs-RGO mass contents. The insets are the optical microscope photographs. As shown in Figure 11, the scratches on all of the samples exposed to IR light almost completely disappear. Comparing Figure 11b–i with Figure 11a, it can be seen that the surface of the composite film is dense and smooth. According to the scratch healing theory, the scratch healing process of polymer mainly includes the wetting, diffusion, rearrangement of polymer chains, and formation of a semi-interpenetrating network structure through the interpenetrating bridge of bonds in the crack section [40]. For DA-PU materials, in addition to the steps mentioned above, the scratch healing process also includes DA reaction and retro-DA (rDA) reaction. That is, the covalently cross-linked networks demonstrate reversibility at elevated temperatures via retro-DA reaction, which is the cleavage of DA. After cooling down to 60 °C and continued heating for a certain time, the broken links are able to form an interconnection with the sample. Figure 12 shows a schematic diagram of the self-healing of the composite film under IR light.

A certain high temperature and a long time are conducive to the cleavage of DA bonds, which is better for the diffusion of DA monomers into the scratch and the formation of interconnections to fill the scratch. The DA reaction and rDA reaction can be repeated many times by treating materials at different temperatures. In our experiment, it was seen that the long PU-DA chains break into furan-terminated prepolymer and BMI gradually because of the rDA reaction at elevated temperatures. The resultant short chains and molecules can move and diffuse from one side to another side of a scratch under thermal action. As a result, a scratch in the composite film can be filled by polymer chains and BMI. When further treated at 60 °C, the DA reaction between furan-terminated prepolymer and BMI takes place again, and the long PU-DA chains regenerate. Due to the low temperature rise of pure DA-PU film under IR light, it takes a long time to achieve the complete disappearance of scratches. Due to the excellent IR absorption and energy conversion and transfer of the AgNWs-RGO, the temperature of the composite film rises quickly under IR light, which makes the scratch disappear completely in a short time. However, as seen in Figure 8, under infrared light, an increase in the mass content of AgNWs-RGO in the composite film does not lead to a higher temperature of the composite film. In addition, it should be noted that we also measured the resistivity of the composite film before and after healing (the scratch position); there was little difference (excluding error factors), and the film still maintained good conductivity.

A small amount of AgNWs-RGO (5 wt% and 10 wt%) is beneficial in reducing the self-healing time of the scratches on the surface of the composite. As the mass content of AgNWs-RGO increases, the self-healing time of the scratch on the surface of the composite is increased. As mentioned above, the IR absorption and thermal energy conversion of the AgNWs-RGO can prompt the temperature of the composite film to rise rapidly. However, the AgNWs-RGO in the DA-PU matrix can hinder the free movement and the rotation of the DA-PU molecular chain, which is not conducive to the DA reaction and maintaining the intrinsic flexibility of the DA-PU matrix. With the appropriate AgNWs-RGO content, the good compatibility between AgNWs-RGO and DA-PU is helpful in reducing the hindrance of AgNWs-RGO to the free movement of DA-PU molecular chain, and the IR thermal response of AgNWs-RGO is obvious, which is conducive to the DA reaction. However, when the AgNWs-RGO content is high, the aggregates of AgNWs-RGO reduce the IR thermal response of the AgNWs-RGO and hinder the movement of DA molecular chains, and with the low content of the DA-PU in the composite film, there is not enough free movement, wetting, and scratch filling of the DA-PU molecular chain [18,19,20,21]. In addition, we also found that the temperatures of the composite films under the above-mentioned IR illumination time are about 109.5 °C, 162.4 °C, 165 °C, 173.2 °C, 178.5 °C, 167 °C, 154.7 °C, 146 °C, and 139.7 °C for the AgNWs-RGO mass contents in the composite film of 0 wt%, 5 wt%, 10 wt%, 15 wt%, 20 wt%, 25 wt%, 30 wt%, 35 wt%, and 37 wt%, respectively, indicating that the increase in the temperature of the composite film does not result in a better self-healing effect of the composite film but that the content of the DA-PU matrix in the composite film plays a key role in the self-healing effect. Therefore, this phenomenon also demonstrates that the self-healing behavior of the DA-PU depends on the thermo-reversible DA reaction and the thermal movement of molecular chains [54].

**Figure 13:**
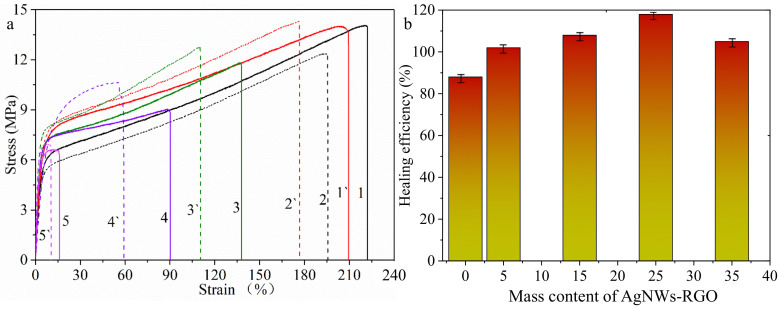
Stress–strain curves (**a**) of composite films with different AgNWs-RGO mass contents before (1–5) and after (1′–5′) healing and self-healing efficiencies (**b**) of the composite films. (1, 1′) 0 wt%; (2, 2′) 5 wt%; (3, 3′) 15 wt%; (4, 4′) 25 wt%; (5, 5′) 35 wt%.

To further quantitatively evaluate the self-healing behavior of the scratch of the composite film, we chose the composite films with AgNWs-RGO contents of 5 wt%, 15 wt%, 25 wt%, and 35 wt% to measure the tensile strength of the composite films before and after self-healing and calculate the self-healing efficiency. Figure 13 shows the stress–strain curves (Figure 13a) and the self-healing efficiency (Figure 13b) of the composite film. The curves in Figure 11a of (1, 1′), (2, 2′), (3, 3′), (4, 4′), and (5, 5′) represent the stress–strain of the composite films with AgNWs-RGO contents of 0 wt%, 5 wt%, 15 wt%, 25 wt%, and 35 wt%, respectively, before and after self-healing. It can be observed that the fracture strain of the healed sample displays an obvious decrease, and the decrease degree of the fracture strain increases as the mass content of AgNWs-RGO in the composite film increases from 0 to 25 wt%, indicating that the AgNWs-RGO impacts the flexibility of the composite film. The tensile strength of the composite film is obviously higher than that before self-healing, meaning full self-healing performance. The tensile strength of the DA-PU film decreases after self-healing. As demonstrated in Figure 13b, the self-healing efficiencies of the pure DA-PU and the composite films with AgNWs-RGO contents of 5 wt%, 15 wt%, 25 wt%, and 35 wt%, are 88%, 102%, 108%, 118%, and 105% respectively, indicating that the AgNWs-RGO improves the IR thermal self-healing behavior of the composite film.

As mentioned above, due to the rDA reaction, the long DA-PU chains break into short polymer segments, and then the short polymer segments move, diffuse, and fill the scratch of the sample at elevated temperature under IR light. After further heat treatment at 60 °C, the DA reaction between the furan-terminated prepolymer and BMI occurs again and the long PU-DA chain is regenerated. Thus, scratch is re-mended by the filled chains and the strength of the sample is restored. However, not all of the furan-terminated prepolymer and BMI are involved in DA reactions, only most of them, so the strength of the pure DA-PU film declines [55,56]. The reasons for the reinforcement of the tensile strength of the composite film after healing are as follows: (1) At elevated temperature, the DA-PU undergoes melting and the rDA reaction, breaking into short polymer segments; the adhesion interaction between the AgNWs-RGO via RGO and the polymer matrix is enhanced through the existence of the hydroxyl and carboxyl groups on the edge of the GO. (2) The orientation alignment and slip of the AgNWs-RGO at the fracture site require additional energy. (3) During the melting process of DA-PU at elevated temperature, the distribution of the AgNWs-RGO in the DA-PU matrix maybe be improved, so the possibility of stress concentration is reduced. (4) The excellent IR thermal effect of AgNWs-RGO causes the temperature of the composite film to rise rapidly to high temperature, which leads to more DA-PU melting behavior and rDA reactions [49,50,51,52,53,54].

**Figure 14:**
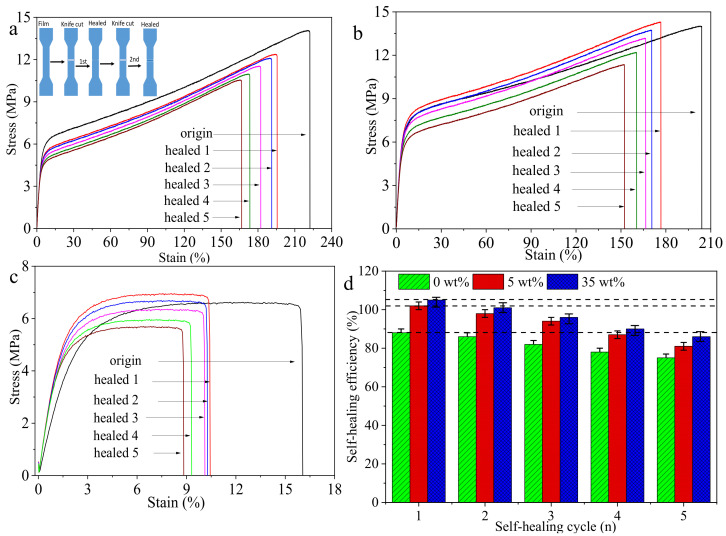
Stress–strain curves of the composite films with AgNWs-RGO contents of 0 wt% (**a**), 5 wt% (**b**), and 35 wt% (**c**) and the self-healing efficiencies of three samples for five cycles (**d**). The inset is a schematic diagram of the scratch self-healing cycle.

To explore the multiple self-healing ability of the composite film, we chose the composite films with the AgNWs-RGO contents of 0 wt%, 5 wt%, and 35 wt% for five self-healing cycles under IR light. Figure 14 shows the stress–strain curves of the composite films with AgNWs-RGO contents of 0 wt% (Figure 14a), 5 wt% (Figure 14b), and 35 wt% (Figure 14c) and the self-healing efficiency of five self-healing cycles of three samples. The inset in Figure 14a is a schematic diagram of the scratch self-healing cycle. As the number of self-healing cycles increases, the fracture strain of samples decreases, indicating that the flexibility of the films gradually decreases. The self-healing efficiencies of the pure DA-PU film and the composite films with the AgNWs-RGO contents of 5 wt% and 35 wt% are 78%, 87%, and 90% after four self-healing cycles and then decrease to 75%, 81%, and 86% after five self-healing cycles, respectively. This indicates that the AgNWs-RGO improves the multiple IR thermal self-healing ability of the composite film and the composite film exhibits excellent self-healing performance. Moreover, the higher the AgNWs-RGO mass content in the composite film, the better the multiple self-healing performance.

As mentioned above, the self-healing behavior of the DA-PU depends on the thermo-reversible DA reaction and the thermal movement of molecular chains. However, with the cycle of the self-healing behavior, some DA bonds and DA adducts do not regenerate every time; at the same time, the self-polymerization of maleimide may occur, forming irreversible bonds and resulting in a gradual decrease in the self-healing efficiency of the film. However, the excellent IR thermal effect of the AgNWs-RGO can promote the melting and rDA reaction of DA-PU, and at the same time, the good mechanical properties of the AgNWs-RGO synergistically enhance the mechanical properties of composite film, so the self-healing efficiency of the composite film after healing cycles is still higher than that of the pure DA-PU.

**Figure 15:**
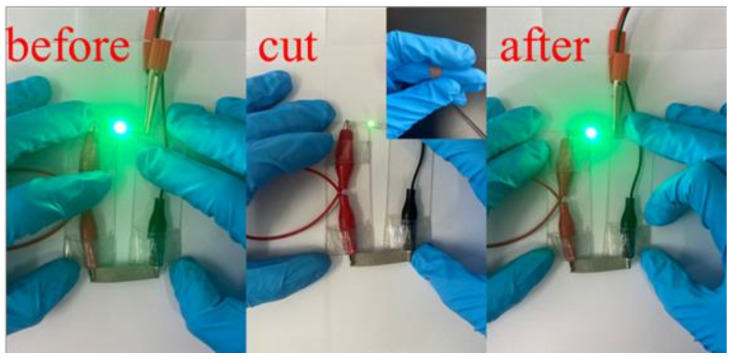
Photographs of LED bead on the composite film before healing, after the cut, and after healing under near-IR irradiation.

To examine the application of the self-healing conductive composite film, we chose the composite film with AgNWs-RGO mass content of 30 wt%, which has good electrical conductivity, to form a circuit with a 0.5 W light-emitting diode (LED) bead. Figure 15 shows the photographs of the LED bead on the composite film before healing, after the cut, and after healing under IR irradiation. When the film was not damaged, the LED bead displayed a bright and dazzling light. When a scratch was made on the composite film surface with a knife, the LED light was dimmed. After the healing process, the LED bead again displayed a bright and dazzling light, indicating that the damaged conductive networks had been repaired.

## 4. Conclusions

We synthesized the hybrid composite of AgNWs-RGO in situ by an improved polyol–thermal method. DA-PU of furan and maleimide moieties was prepared. AgNWs-RGO with a mass content of 5–37 wt% was introduced into the DA-PU matrix to prepare self-healing composite conductive films. The experimental results demonstrate that the AgNWs-RGO endows the DA-PU film with good electrical and thermal conductivity and infrared thermal response ability, while the mechanical properties of the DA-PU film decrease as the AgNWs-RGO mass content increases. The resistivities of the composite films with AgNWs-RGO mass contents of 25 wt% and 37 wt% are 0.544 Ω·m and 0.005 Ω·m, respectively. As the mass content of AgNWs-RGO increases from 5 wt% to 35 wt%, the thermal conductivity of the composite film increases by a factor of approximately 2.2, and a significant decrease in the tensile strength and strain of the composite film and an increase in Young’s modulus of the composite film are observed. The AgNWs-RGO remarkably improves the IR response performance of the composite film. The scratches of the pure DA-PU and the composite films with AgNWs-RGO mass contents of 5 wt%, 10 wt%, 15 wt%, 20 wt%, 25 wt%, 30 wt%, 35 wt%, and 37 wt% disappear after 300 s, 90 s, 100 s, 460 s, 540 s, 600 s, 870 s, 1205 s, and 1515 s, respectively, under infrared light irradiation. The self-healing efficiencies of the pure DA-PU and the composite films with AgNWs-RGO mass contents of 5 wt%, 15 wt%, 25 wt%, and 35 wt% are 88%, 102%, 108%, 118%, and 105% respectively. Multiple self-healing cycles of the composite films with AgNWs-RGO contents of 0 wt%, 5 wt%, and 35 wt% under IR light irradiation show that the self-healing efficiencies are 78%, 87%, and 90%, respectively, after four self-healing cycles and decrease to 75%, 81%, and 86% after five self-healing cycles, respectively, indicating that the AgNWs-RGO improves the multiple IR thermal self-healing ability of the composite film.

## Figures and Tables

**Table 1:** Mechanical properties of the composite films.

AgNWs-RGO Mass Content (wt%)	Stress at Break (MPa)	Young’s Modulus (MPa)	Strain at Break (%)
0 wt%	14.08	19.30	221.94
5 wt%	13.27	26.93	209.26
10 wt%	12.87	27.37	232.70
15 wt%	11.84	140.62	138.01
20 wt%	10.35	238.91	61.30
25 wt%	9.05	192.39	90.38
30 wt%	7.54	202.84	33.24
35 wt%	6.63	233.07	15.85
37 wt%	10.55	246.40	23.03

## Data Availability

Data is contained within the article.

## Supplementary Materials

The following supporting information can be downloaded at: https://www.mdpi.com/article/10.3390/membranes12040405/s1, Figure S1: SEM images of products of the reaction of solutions with the GO:AgNO_3_ mass content ratios of 1:98.5 (a), 1:48.9 (b), 1:32.4 (c), 1:15.7 (d), 1:10.1 (e) and 1:7.3 (f) at 150 °C for 3 h. The insets are photos of the solution after the reaction; Figure S2: SEM images of products of the reaction of solutions with the GO:AgNO_3_ mass content ratio of 1:32.3 at 150 °C (a), 160 °C (b), and 170 °C (c) for 4 h and 5 h; Figure S3: IR spectroscopy monitored during the reaction progress; Figure S4: Local magnification of Figure S3; Figure S5: Gel permeation chromatogram of DA-PU. The inset is a sample photo.
