# Supplementary material for: Self-Healing Silver Nanowires and Reduced Graphene Oxide/Polyurethane Composite Film Based on the Diels–Alder Reaction under Infrared Radiation
Source: Membranes (Basel). 2022 Apr 6;12(4):405. doi: 10.3390/membranes12040405 (PMC9030009; doi:10.3390/membranes12040405)
Supplement: Supplementary file 1 [file membranes-12-00405-s001.zip › membranes-1650649-supplementary.pdf]

Self-Healing Silver Nanowires and Reduced Graphene Oxide/Polyurethane Composite Film Based on the Diels–Alder Reaction under Infrared Radiation

Yi Wang, Zhimin Zhou, Jiali Chen, Sixing Li, Han Zheng, Jiaxin Lu, Shuyue Wang, Jiahao Zhang, Kaiwen Lin, Ke Wang and Yuehui Wang *

Materials and Food Department, Zhongshan Institute, University of Electronic Science and Technology of China, Zhongshan 528402, Guangdong, China; wangyi@zsc.edu.cn (Y.W.); zzmzsedu@126.com (Z.Z.); chejiali0104@126.com (J.C.); 2019040801083@stu.zsc.edu.cn (S.L.); 2019040801059@stu.zsc.edu.cn (H.Z.); 2019040802023@stu.zsc.edu.cn (J.L.); 2019040802028@stu.zsc.edu.cn (S.W.); zjhzsedu@126.com (J.Z.); kevinlin1990@163.com (K.L.); wkzsedu@126.com (K.W.)

* Correspondence: wangzsedu@126.com; Tel.: +86-760-8832-5402

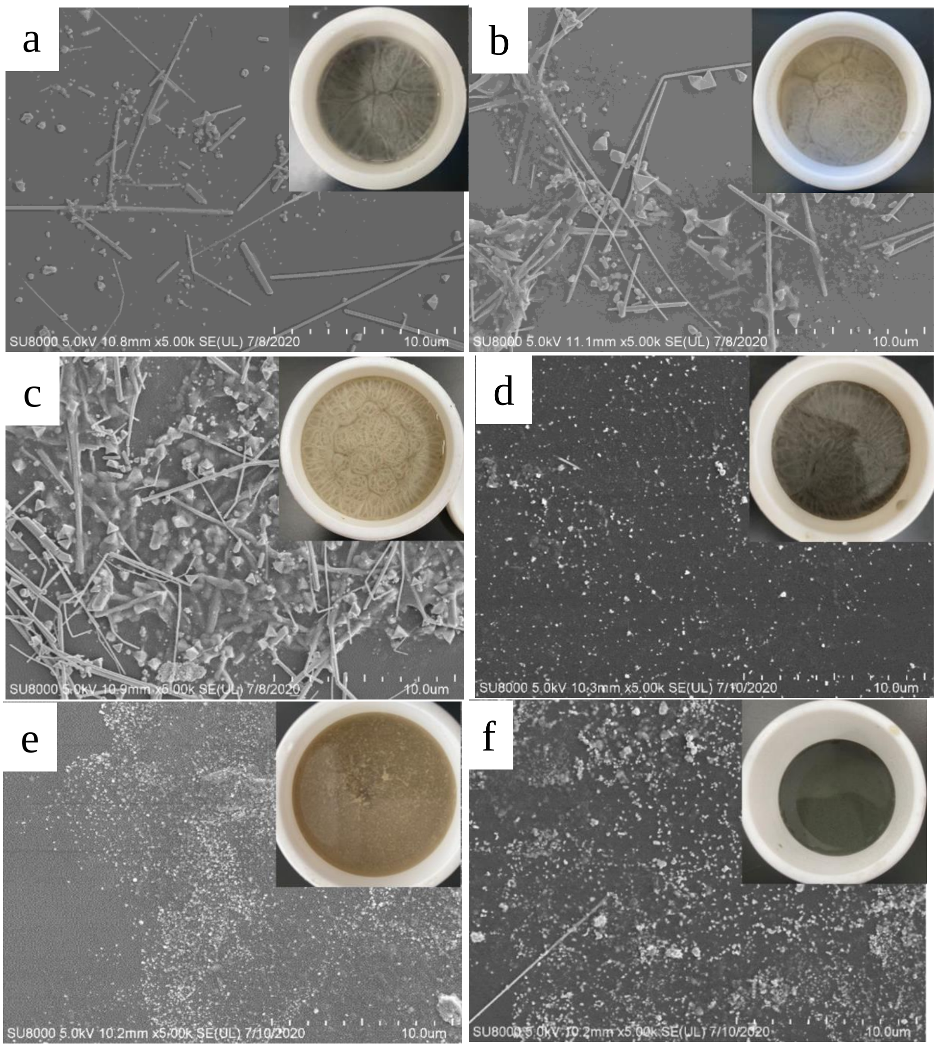

Figure S1. SEM images of products of the reaction solutions of the ratio of mass content of GO:$AgNO_3$ of 1:98.5 (**a**), 1:48.9 (**b**), 1:32.4 (**c**), 1:15.7 (**d**), 1:10.1 (**e**) and 1:7.3 (**f**) at 150 ℃ for 3 h, respectively. The inserts are photos of the solution after the reaction.

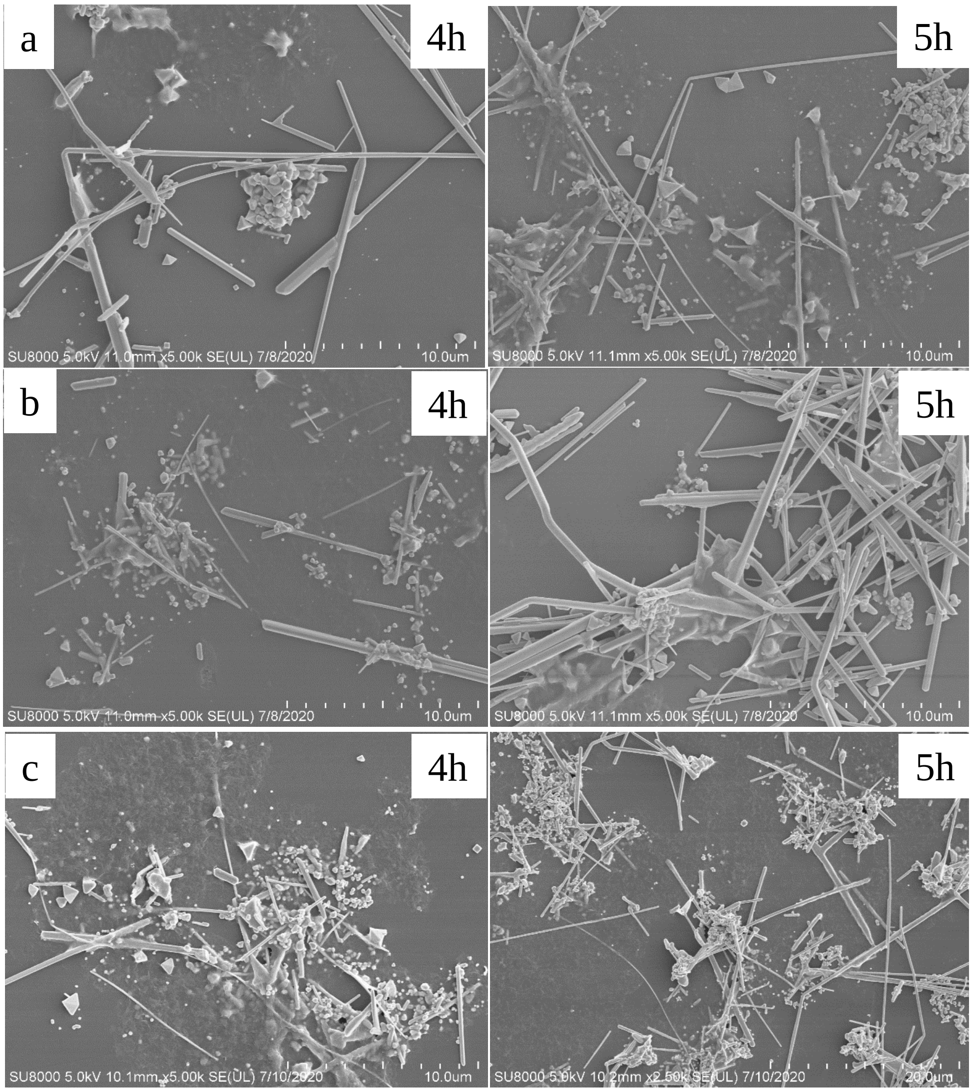

Figure S2. SEM images of products of the reaction solutions of the ratio of mass content of GO:$AgNO_3$ of 1:32.3 at 150 ℃ (**a**)、160 ℃ (**b**), and 170 ℃(**c**) for 4 h and 5 h, respectively.

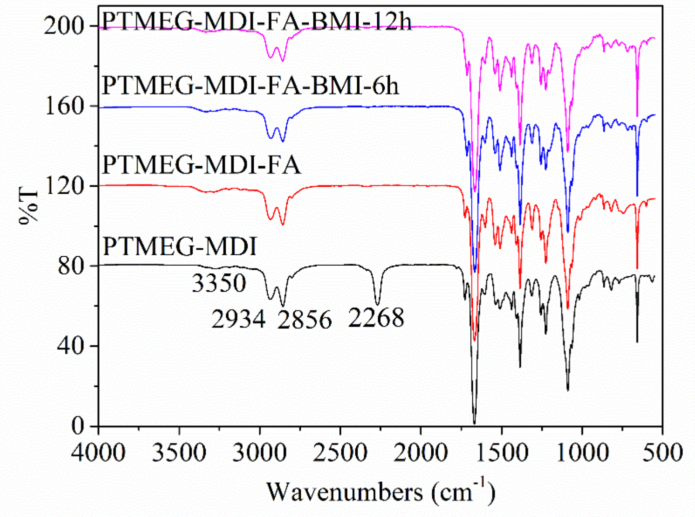

Figure S3. IR-spectroscopy monitored during the reaction progress.

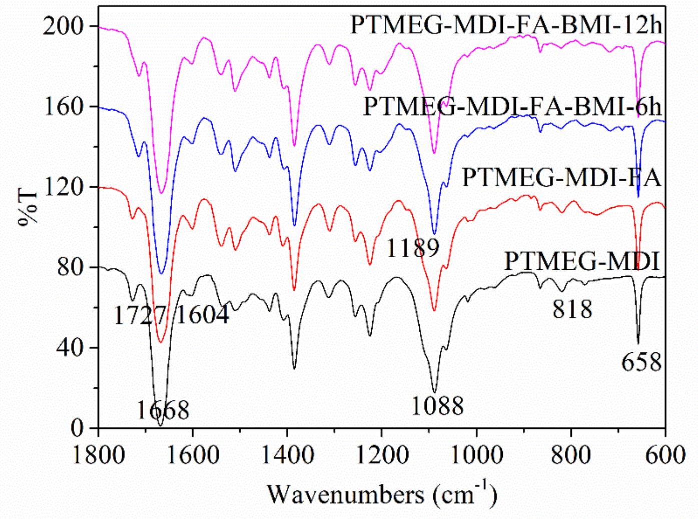

Figure S4. Local magnification of Figure S3.

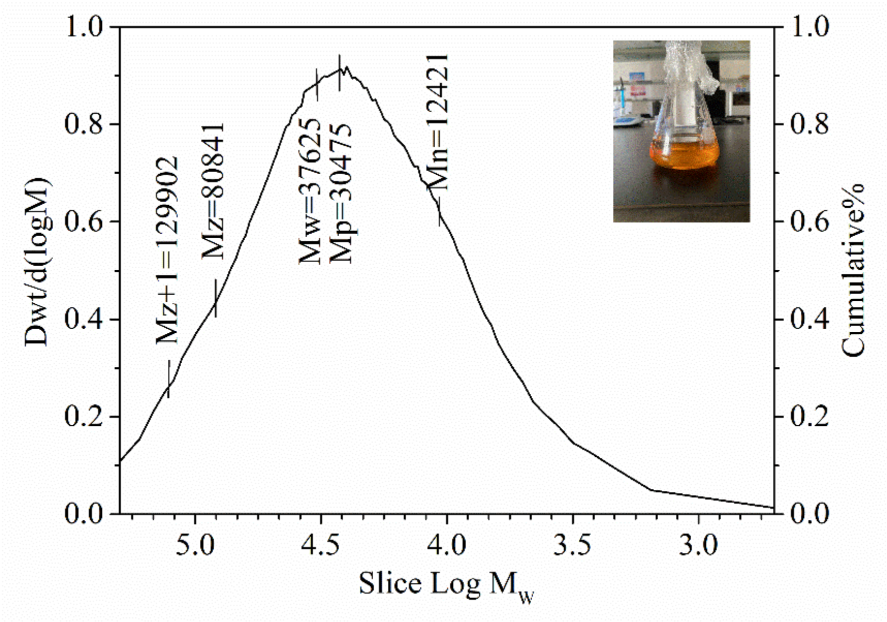

Figure S5. Gel permeation chromatogram of DA-PU. The insert is sample photo.
